# Mortality after major bleeding in Asian atrial fibrillation patients receiving different direct oral anticoagulants: a nationwide, propensity score study

**DOI:** 10.1038/s41598-024-55500-z

**Published:** 2024-02-27

**Authors:** Jiun-Hao Yu, Pei-Ru Li, Dong-Yi Chen, Wen-Kuan Huang, Lai-Chu See

**Affiliations:** 1 Department of Emergency Medicine, China Medical University Hsinchu Hospital, China Medical University, Hsinchu, Taiwan; 2 Graduate Institute of Management, Chang Gung University, Taoyuan City, Taiwan; 3 Department of Public Health, College of Medicine, Chang Gung University, 259, Wenhua 1st Rd., Guishan Dist., Taoyuan City, 33302 Taiwan; 4 Division of Cardiology, Department of Internal Medicine, Chang Gung Memorial Hospital at Linkou, Taoyuan City, Taiwan; 5 School of Medicine, College of Medicine, Chang Gung University, Taoyuan City, Taiwan; 6 Division of Hematology/Oncology, Department of Internal Medicine, Chang Gung Memorial Hospital at Linkou, Taoyuan City, Taiwan; 7 Division of Rheumatology, Allergy, and Immunology, Chang Gung Memorial Hospital at Linkou, Taoyuan City, Taiwan; 8 Biostatistics Core Laboratory, Molecular Medicine Research Center, Chang Gung University, Taoyuan City, Taiwan

**Keywords:** Therapeutics, Cardiovascular biology, Drug safety, Pharmacology, Health care

## Abstract

In this research, we assessed mortality after major bleeding events in atrial fibrillation (AF) patients taking four direct oral anticoagulants (DOACs). Drawing data from the Taiwan National Health Insurance Research Database between 2016 and 2019, we focused on AF patients on DOACs who had major bleeding episodes. Using propensity score stabilized weighting, we established four comparable pseudo-DOAC groups. Among 2770 patients (460 dabigatran, 1322 rivaroxaban, 548 apixaban, 440 edoxaban), 85.3% were prescribed low-dose regimens. The 7-day mortality rate was 9.0%, surging to 16.0% by the 30th day. Compared with dabigatran, there was a distinct divergence in 7-day mortality of factor Xa inhibitors (*p* = 0.012), with hazard ratios of 1.83 (95% CI 1.11–3.00, *p* = 0.017) for rivaroxaban, 2.13 (95% CI 1.23–3.66, *p* = 0.007) for apixaban, and 2.41 (95% CI 1.39–4.19, *p* = 0.002) for edoxaban. This pattern remained consistent when analyzing the subgroup that received lower dosages of DOACs. In conclusion, factor Xa inhibitors were associated with a significantly higher risk of 7-day mortality following major bleeding events than dabigatran among AF patients.

## Introduction

In atrial fibrillation (AF) patients, direct oral anticoagulants (DOACs) have been associated with comparable or even lower risks of stroke, major bleeding, and mortality compared to warfarin^1,2^. Further, a meta-analysis of randomized trials highlighted that all four DOAC types, including dabigatran, rivaroxaban, apixaban, and edoxaban, exhibit a better safety profile than warfarin^3^. Despite the lower bleeding risk, managing such events remains challenging for physicians in the emergency department (ED)^4^. Therefore, low-dose DOACs are frequently prescribed in real-world clinical practice^5–7^, reflecting physicians' concerns about major bleeding.

While extensive research has compared the safety profiles of DOACs to warfarin, the safety profile in terms of short-term mortality due to major bleeding among different DOACs remains limited^8^. Furthermore, most of these studies have focused on the incidence of major bleeding in AF patients, rather than short-term mortality following bleeding events^9–11^. Hence, we used the Taiwan National Health Insurance Research Database for a nationwide cohort study. We aimed to compare the short-term mortality after major bleeding in Asian patients with AF and receiving four DOACs (dabigatran, rivaroxaban, apixaban, and edoxaban).

## Methods

### Data source

The primary data sources are the Taiwan National Health Insurance Research Database (NHIRD) and Taiwan Death Registry, available in the Health and Welfare Data Science Center. All the datasheets have been linked with individual personal identification numbers (PINs) encrypted for research. To avoid any disclosure of patient identity, the center reviews all analyzed results before releasing them^12^. The Institutional Review Board of China Medical University Hospital, Taiwan, approved this study and waived informed consent (CMUH109-REC2-146). All methods were performed in accordance with the relevant guidelines and regulations. Our study was conducted in compliance with Good Clinical Practice guidelines and adhered to the ethical standards outlined in the Declaration of Helsinki.

### Study design

**Figure 1:**
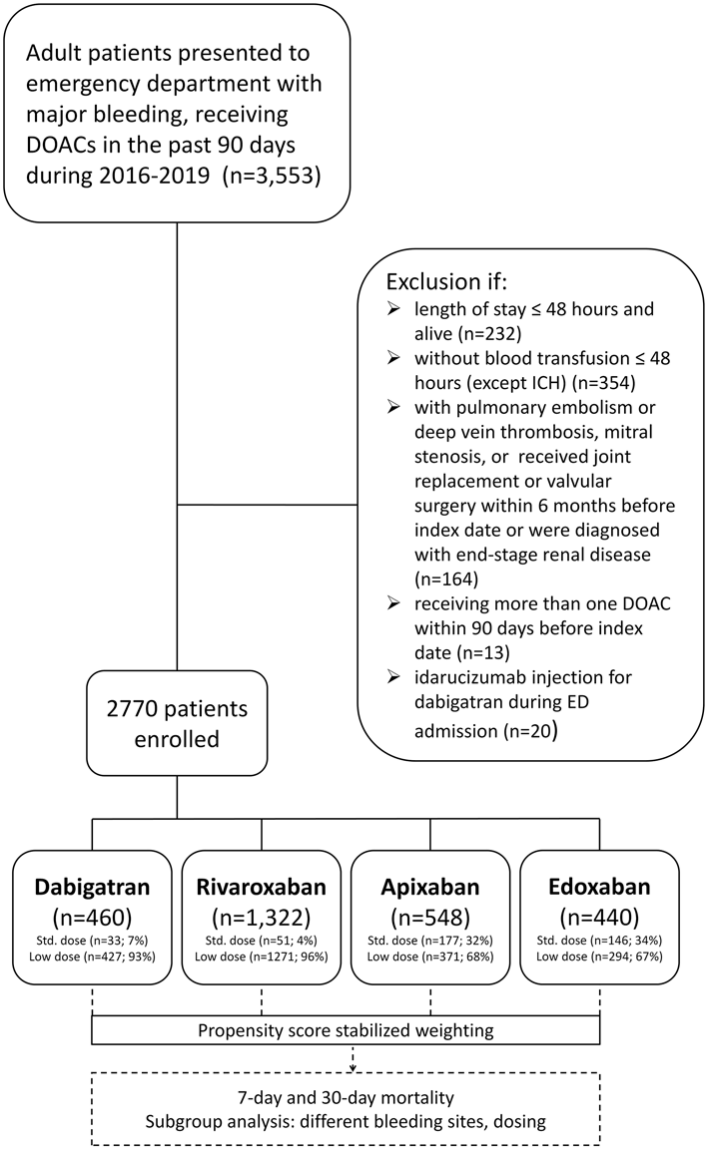
Patient Enrollment Flowchart. This figure details the enrollment process for the study involving atrial fibrillation patients treated with dabigatran, rivaroxaban, apixaban, and edoxaban (DOACs, direct oral anticoagulants; ED, emergency department; ICH, intracranial hemorrhage; std., standard).

In Taiwan, dabigatran was first approved on June 1, 2012, followed by rivaroxaban (February 1, 2013), apixaban (June 1, 2014), and edoxaban (September 1, 2016), respectively. This nationwide retrospective cohort included patients with AF who received one of the DOACs and experienced following major bleeding events and visited ED during 2016–2019. Major bleeding events included intracranial hemorrhage (ICH), gastrointestinal (GI) bleeding, and bleeding at other critical sites. Noted that some patients had more than one bleeding sites. Please refer to the Supplementary Table S1 for International Classification of Diseases (Ninth and Tenth Revisions) Clinical Modification (ICD-9-CM and ICD-10-CM) codes used to define major bleeding. The index date is the ED visit for major bleeding management. We excluded patients: (1) those aged < 18 years old, (2) mild bleeding events, which did not need blood transfusion within 48 h after the ED visit except for ICH, or were discharged and alive within 48 h after the ED visit, (3) pulmonary embolism or deep vein thrombosis, mitral stenosis, or received a joint replacement or valvular surgery within 6 months before ED visit, (4) end-stage renal disease, (5) receiving more than one DOAC type within 90 days before the index date or switched from one DOAC to another before ED visit to reduce misclassification, (6) receiving idarucizumab for reversal of dabigatran during ED visit because the reversal agents for those on Factor Xa inhibitors were unavailable in Taiwan (Fig. 1). We assessed the use of DOACs which included dabigatran, rivaroxaban, apixaban, and edoxaban. The investigated dosages comprised either the standard-dose regimen or the low-dose regimen. Guided by the Taiwan NHIRD for AF treatment, the standard-dose regimen is specified as follows: 150 mg of dabigatran twice daily, 20 mg of rivaroxaban once daily, apixaban 5 mg twice daily, and 60 mg of edoxaban once daily. Patients who were administered dosages below these specified standard doses were categorized into the low-dose group.

In this study, we utilized the coding system defined in the Taiwan National Health Administration to identify surgical and medical hemostasis approaches employed in managing major bleeding events. These approaches are detailed in Supplementary Table S2.

### Study outcomes

The primary outcome of this study was all-cause mortality within the first 7 and 30 days following ED visit due to major bleeding events. The 7-day mark was selected for its criticality in evaluating immediate mortality risk^13,14^. Evidence suggests that the risk of death is particularly high in the days immediately following ED admission, increasing cumulatively over time^15^. Notably, research indicates that approximately half of all deaths following major bleeding events occur within the first month, with a significant proportion—around one-third—occurring on the first day itself^8^. Consequently, the 7-day period provides vital insight into the acute phase of mortality risk. On the other hand, the 30-day timeframe was selected to offer a broader view of mortality outcomes, which is especially pertinent in conditions like intracranial hemorrhage where longer-term risks are relevant^8,16,17^.

### Covariates

The covariates were relevant comorbidities or medications prior to the index date. The CHA2DS2-VASc score (congestive heart failure, hypertension, age 75 years or older, diabetes, previous stroke/transient ischemic attack, vascular disease, age 65–74 years, female) had been derived to predict the risk of thromboembolism in patients with AF^18^. The HAS-BLED score (hypertension, abnormal renal or liver function, stroke, bleeding history, labile INR, age 65 years or older, and antiplatelet drug or alcohol use) has been derived to predict the risk of bleeding in AF patients treated with DOACs^19^. Medications were confined to prescription at least once within 3 months before the index date, including NSAIDs (nonsteroidal anti-inflammatory drugs), PPI (proton pump inhibitor), ACEI (angiotensin-converting-enzyme inhibitor)/ARB (angiotensin II receptor antagonists, β-blocker, DHP-CCB (dihydropyridine calcium channel blockers), nonDHP-CCB (diltiazem/verapamil). Please refer to the Supplemental Table S1 for ICD-9-CM and ICD-10-CM codes used to define comorbidities.

### Statistical analysis

We used propensity score stabilized weighting (PSSW) to create four pseudo- DOAC groups with similar baseline covariates^20^. The propensity score (PS) was estimated using the generalized boosted model (GBM) with all covariates in Table 1 included, except for CHA2DS2-VASc and HAS-BLED scores because these two scores were already a combination of other covariates. GBM involves an iterative process with multiple regression trees to capture complex and nonlinear associations to maximize the balance in the covariates across the four DOAC groups^21^. Furthermore, the PS provided by GBM is less affected by model misspecification and outlying weights^22^. We used the maximum of absolute standardized mean difference (max(ASMD)) to assess whether the four DOAC groups were well-balanced in covariates if an ASMD value of ≤ 0.1^23^. We also examined the overlap of the PS distribution for the four DOAC groups because PSSW shows bias and excessive variance in the presence of PS tails^24^.

**Table 1:** Demographic characteristics and comorbidities when admitted to emergency department due to major bleeding among patients with atrial fibrillation and treated with the four direct oral anticoagulants in Taiwan, 2016–2019, after propensity score stabilized weighting.

	Dabigatran (n = 460)	Rivaroxaban (n = 1322)	Apixaban (n = 548)	Edoxaban (n = 440)	Max ASMD
Age, years	80.2 ± 8.2	79.6 ± 9.7	79.9 ± 8.5	80.1 ± 7.9	0.0617
Male	199.19 (52.0%)	680 (53.5%)	246.09 (54.5%)	192.27 (53.1%)	0.0562
CHA_2_DS_2_-VASc	4.8 ± 1.6	4.8 ± 1.7	4.8 ± 1.6	4.8 ± 1.6	0.0258
HAS-BLED score	3.6 ± 1.1	3.6 ± 1.3	3.7 ± 1.2	3.8 ± 1.2	0.0971
History of bleeding	228.05 (59.5%)	746.97 (58.8%)	263.17 (58.3%)	214.64 (59.3%)	0.0266
Hypertension	275.11 (71.8%)	895.35 (70.5%)	323.49 (71.7%)	263.63 (72.8%)	0.0541
Hyperlipidemia	159.32 (41.6%)	534.32 (42.1%)	188.98 (41.9%)	151.93 (42.0%)	0.0106
Cardiovascular disease	254.46 (66.4%)	837.2 (65.9%)	303.33 (67.2%)	233.72 (64.5%)	0.0622
Stroke	168.78 (44.1%)	576.16 (45.4%)	205.85 (45.6%)	158.18 (43.7%)	
Congestive heart failure	75.25 (19.6%)	205.63 (16.2%)	94.45 (20.9%)	58.47 (16.1%)	
Chronic ischemic heart disease	68.77 (17.9%)	239.93 (18.9%)	64.97 (14.4%)	64.11 (17.7%)	
Peripheral arterial disease	3.21 (0.8%)	29.01 (2.3%)	5 (1.1%)	2.97 (0.8%)	
Coronary artery bypass graft	5.1 (1.3%)	21.07 (1.7%)	9.38 (2.1%)	6.52 (1.8%)	
Percutaneous coronary intervention	39.99 (10.4%)	145.34 (111.4%)	40.79 (9.0%)	39.61 (10.9%)	
Diabetes mellitus	162.1 (42.3%)	526.35 (41.5%)	189.81 (42.1%)	148.86 (41.1%)	0.0266
Chronic kidney disease	96.11 (25.1%)	307.53 (24.2%)	111.65 (24.7%)	90.81 (25.1%)	0.0212
Chronic lung disease	51.99 (13.6%)	202.23 (15.9%)	68.64 (15.2%)	57.14 (15.8%)	0.07
Chronic liver disease	30.03 (7.8%)	95.72 (7.5%)	31.24 (6.9%)	25.15 (7.0%)	0.0384
Malignancy	73.14 (19.1%)	276.01 (21.7%)	99.08 (22.0%)	76.64 (21.2%)	0.0781
Use of antiplatelet	89.06 (23.24%)	287.11 (22.6%)	93.88 (20.8%)	87.91 (24.28%)	0.0916
Use of NSAIDs	121.03 (31.6%)	401.28 (31.6%)	147.26 (32.6%)	109.63 (30.3%)	0.056
Use of PPI	225.87 (58.9%)	731.2 (57.6%)	263.92 (58.5%)	208.84 (57.7%)	0.0293
Use of ACEI/ARB	248.28 (64.8%)	811.11 (63.9%)	281.72 (62.4%)	229.58 (63.4%)	0.0539
Use of β-blocker	220.23 (57.5%)	731.45 (57.6%)	262.06 (58.1%)	213.44 (59.0%)	0.0328
Use of dihydropyridine CCB	191.14 (49.9%)	640.16 (50.4%)	232.69 (51.6%)	185.8 (51.3%)	0.0370
Use of diltiazem/verapamil	84.16 (22.0%)	279.09 (22.0%)	97.09 (21.5%)	77.55 (21.4%)	0.0146
Use of statin	131.95 (34.4%)	444.87 (35.0%)	159.97 (35.5%)	134.99 (37.3%)	0.0645
Use of digoxin	66.49 (17.4%)	220.08 (17.3%)	74.61 (16.5%)	63.61 (17.6%)	0.0302

ACEI indicates angiotensin-converting enzyme inhibitor; ARB, angiotensin II receptor antagonists; CHA_2_DS_2_-VASc, congestive heart failure, hypertension, age 75 years or older, diabetes mellitus, previous stroke/transient ischemic attack, vascular disease, age 65–74 years, female; CCB, calcium channel blockers; HAS-BLED, hypertension, abnormal renal or liver function, stroke, bleeding history, age 65 y or older, and antiplatelet drug use; NSAIDs, nonsteroidal anti-inflammatory drugs; PPI, proton pump inhibitor.

The mortality rate was computed as the number of patients dying after ED admission divided by the total number of eligible patients. The Cox proportional hazards regression model was made to show the hazard ratio (HR) with the 95% confidence interval (CI) to show the risk of mortality for DOACs, and the reference was dabigatran. Only the DOAC grouping was included in the Cox model because the 4 DOAC groups were well-balanced in covariates after PSSW. Subgroup analysis was made to examine whether the mortality of various types of major bleeding and dosing (low vs. standard) was similar to the overall group. For each subgroup analysis, we reconducted PSSW to ensure the well-balanced covariates among the 4 DOAC groups. A 2-sided *p* < 0.05 indicated statistical significance. All statistical analyses were performed with SAS, version 9.4 (SAS Institute Inc).

## Results

### Patient characteristics and baseline demographics

From June 1, 2016, to December 31, 2019, our research enrolled patients receiving four DOACs, including dabigatran (460 patients), rivaroxaban (1322 patients), apixaban (548 patients), and edoxaban (440 patients). We initially noticed some heterogeneities between study groups (Supplementary Table S3). However, after performing propensity score weighting across all study groups, these heterogeneities were effectively mitigated, resulting in a well-balanced cohort (ASMD < 0.1) (Table 1).

### Mortality Outcomes between DOACs

**Figure 2:**
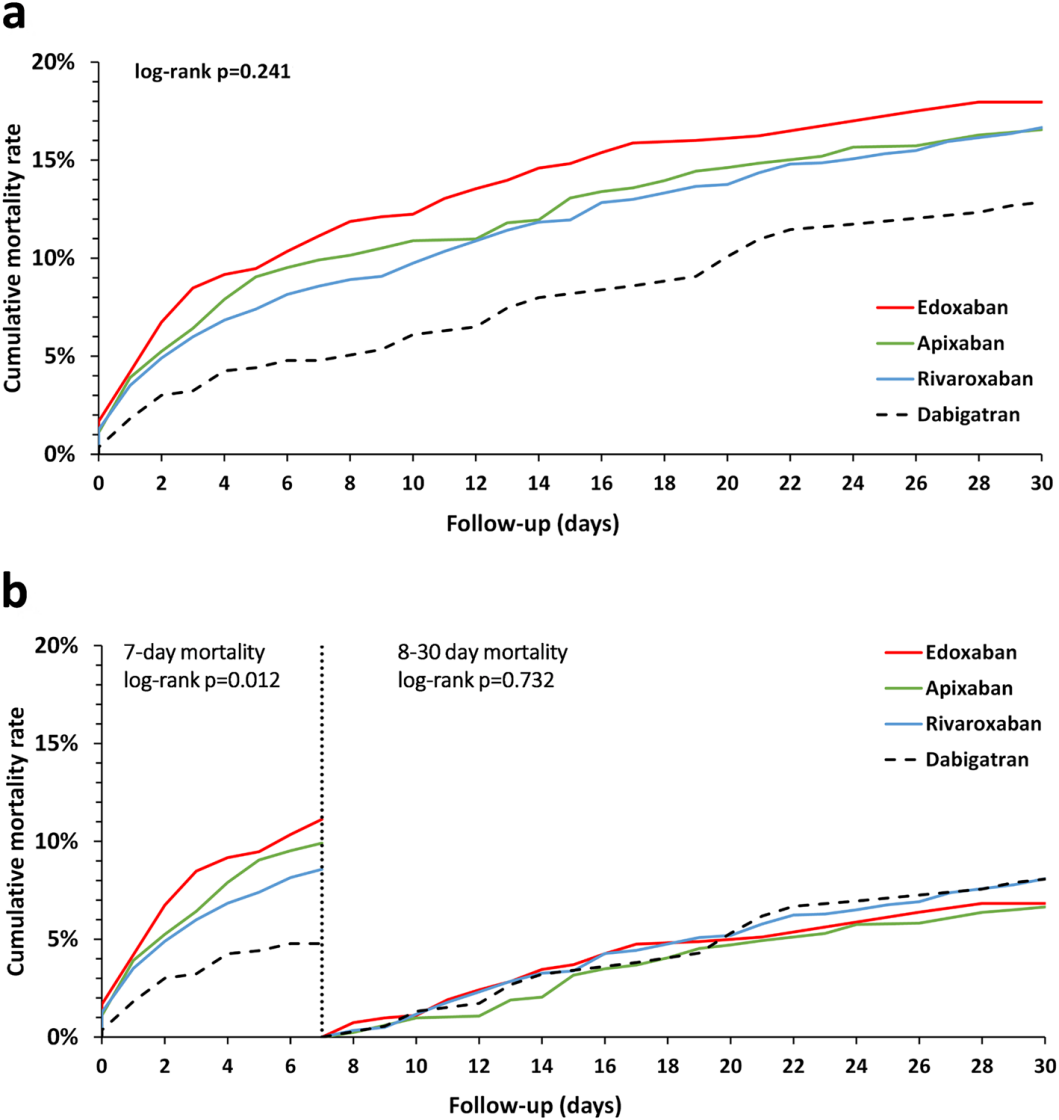
Mortality Rate Comparison Among Four DOACs. (**a**) Illustrates divergent trajectories, though these differences did not achieve statistical significance (log-rank *p* = 0.2411). In (**b**), the observation period is divided into two intervals: the initial 7 days and the subsequent 8–30 days. During the first 7-day interval, a statistically significant separation of mortality rates was observed (log-rank *p* = 0.0115). Conversely, the mortality rates for the 8–30 days period converged, with no statistical significance observed (DOACs, direct oral anticoagulants).

Throughout the 30-day observation period, there was no statistical difference in all-cause mortality across the four DOACs (Fig. 2a, log-rank *p* = 0.241). However, the dabigatran group had lower mortality than those receiving factor Xa inhibitors, including rivaroxaban, apixaban, and edoxaban, during the first 7-day interval (Fig. 2b, log-rank *p* = 0.012). There were no statistically significant differences across different DOAC groups for the 8-30 day interval (log-rank *p* = 0.732).

Table 2 compares mortality rates and hazard ratios among the four DOACs, using dabigatran as the reference group. Before PSSW was implemented, the lowest 7-day mortality rate was observed in the dabigatran group at 4.6%, followed by rivaroxaban at 9.0%, apixaban at 10.8%, and edoxaban at 11.1%. The hazard ratios, which indicate the risk of 7-day mortality, were approximately two times higher for the factor Xa inhibitors than dabigatran. Specifically, after applying PSSW, the hazard ratios were 1.83 (95% CI 1.11–3.00, *p* = 0.017) for rivaroxaban compared to dabigatran, 2.13 (95% CI 1.23–3.66, *p* = 0.007) for apixaban compared to dabigatran, and 2.41 (95% CI 1.39–4.19, *p* = 0.002) for edoxaban compared to dabigatran.

**Table 2:** 7-day mortality rate and hazard ratio before and after propensity score stabilized weighting (PSSW).

	Before PSSW			After PSSW		
	Events	rate	HR (95%CI) (*p* value)	Events	rate	HR (95%CI) (*p* value)
Dabigatran (n = 460)	21	4.6%	Reference	18.32	4.8%	Reference
Rivaroxaban (n = 1322)	119	9.0%	2.02 (1.27–3.21) [0.003]	108.818	8.6%	1.83 (1.11–3.00) [0.017]
Apixaban (n = 548)	59	10.8%	2.44 (1.48–4.01) [0.001]	44.721	9.9%	2.13 (1.23–3.66) [0.007]
Edoxaban (n = 440)	49	11.1%	2.53 (1.52–4.22) [0.001]	40.304	11.1%	2.41 (1.39–4.19) [0.002]

*CI* confidence interval, *HR* hazard ratio.

### Cause of death following major bleeding events

The crude mortality rate was 9% at the 7-day interval and reached 16% at 30 days. In other words, 56% of death occurred during the first 7 days after ED admission, as shown in Table 3. In this initial 7-day period, cerebrovascular disease accounted for 54.4% of the mortality causes, surpassing other causes (26.2%), cardiac disease (12.9%), and cancer (6.5%). In the subsequent 8–30 day period, the proportion of deaths attributable to cerebrovascular disease decreased to 36.9%, followed by other causes (31.3%), cardiac disease (16.9%), and cancer (14.9%). Patients treated with Dabigatran had lower cerebrovascular-related mortality proportion (23.8% within 7 days and 37.5% in the 8–30 day interval) in comparison to those receiving factor Xa inhibitors, though these findings did not reach statistical significance.

**Table 3:** Cause of death at 7-day, 8–30 day, and 30-day intervals following major bleeding events in patients treated with direct oral anticoagulants.

	Total					
	(n = 2770)	Dabigatran (n = 460)	Rivaroxaban (n = 1322)	Apixaban (n = 548)	Edoxaban (n = 440)	*p* value^a^
7-day mortality, events (rate)	248 (9.0%)	21 (4.6%)	119 (9.0%)	59 (10.8%)	49 (11.1%)	
Cerebrovascular disease (%)	54.4%	23.8%	58.0%	54.2%	59.2%	0.1831
Cardiac disease (%)	12.9%	19.0%	11.8%	11.9%	14.3%	
Cancer (%)	6.5%	4.8%	6.7%	5.1%	8.2%	
Others (%)	26.2%	52.4%	23.5%	28.8%	18.4%	
8–30 day mortality, events (rate)	195 (7.0%)	32 (6.9%)	98 (7.4%)	35 (6.4%)	30 (6.8%)	
Cerebrovascular disease (%)	36.9%	37.5%	42.9%	34.3%	20.0%	0.6448
Cardiovascular disease (%)	16.9%	18.8%	15.3%	20.0%	16.7%	
Cancer (%)	14.9%	12.5%	15.3%	14.3%	16.7%	
Others (%)	31.3%	31.3%	26.5%	31.4%	46.7%	
0–30 day mortality, events (rate)	443 (16.0%)	53 (11.5%)	217 (16.4%)	94 (17.2%)	79 (17.9%)	
Cerebrovascular disease (%)	46.7%	32.1%	51.2%	46.8%	44.3%	0.5185
Cardiovascular disease (%)	14.7%	18.9%	13.4%	14.9%	15.2%	
Cancer (%)	10.2%	9.4%	10.6%	8.5%	11.4%	
Others (%)	28.4%	39.6%	24.9%	29.8%	29.1%	

^a^Chi-square test.

### Subgroup analysis of mortality outcomes among different bleeding sites and dose-regimen

**Figure 3:**
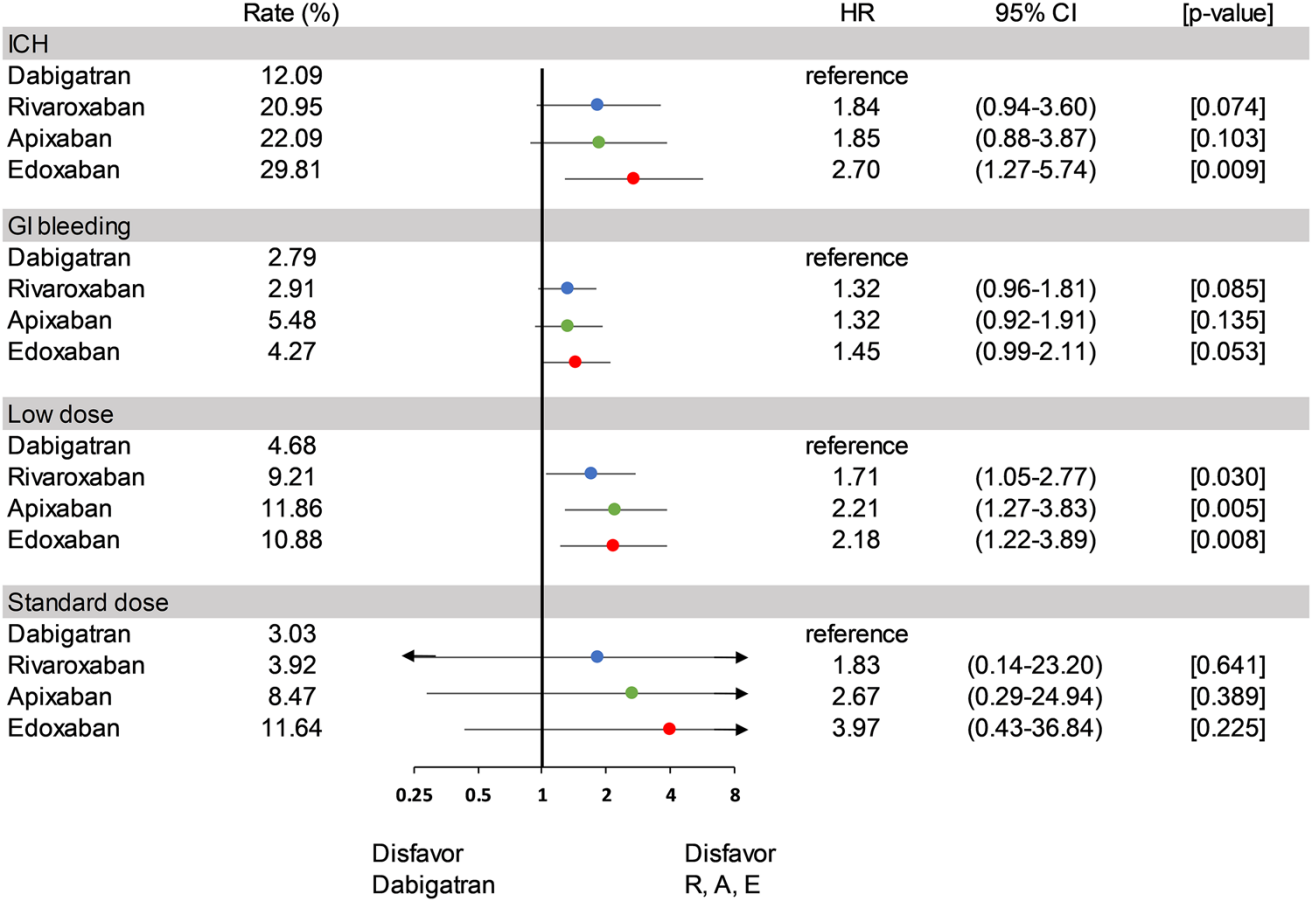
Subgroup Analysis of Cumulative 7-Day Mortality Rates by Bleeding Sites and Dosage. Among patients who experienced ICH, those treated with dabigatran had a 7-day mortality rate of 12.09%, the lowest among the group. For patients on a low-dose regimen, Factor Xa inhibitors exhibited higher hazard ratios for 7-day mortality compared to dabigatran. (DOACs, direct oral anticoagulants; ICH, intracranial hemorrhage; GI, gastrointestinal; R, rivaroxaban; A, apixaban; E, edoxaban; HR, hazard ratio; CI, confidence interval).

Figure 3 displays a subgroup analysis highlighting 7-day mortality rates across different bleeding sites. Among patients who experienced ICH, those treated with dabigatran demonstrated the lowest 7-day mortality rate of 12.09%. In comparison, the rates were 20.95% for rivaroxaban (HR 1.84, 95% CI 0.94–3.60, *p* = 0.074), 22.09% for apixaban (HR 1.85, 95% CI 0.88–3.87, *p* = 0.103), and 29.81% for edoxaban (HR 2.70, 95% CI 1.27–5.74, *p* = 0.009). The only statistically significant difference among these comparisons was observed between dabigatran and edoxaban. Regarding GI bleeding, there was no statistically significant difference in the 7-day mortality rates among the four DOACs.

**Figure 4:**
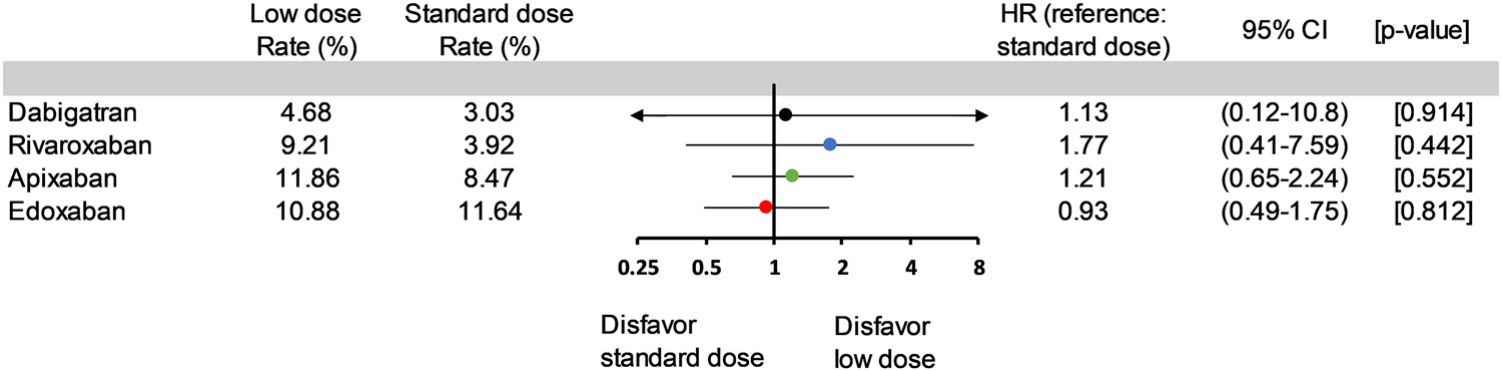
Comparison of Mortality Rates: Standard vs. Low-dose DOACs. Upon examining individual DOACs, there was no significant difference in mortality rates between their standard and low-dose regimens. (DOACs, direct oral anticoagulants; HR, hazard ratio; CI, confidence interval).

Overall, 14.7% of the patients received standard-dose DOACs, whereas 85.3% received low-dose DOACs. The proportion of patients who received low-dose DOACs was 93% in the dabigatran group, 96% in the rivaroxaban group, 68% in the apixaban group, and 67% in the edoxaban group (Fig. 1). Low-dose regimens of four DOACs exhibit considerable disparities in outcomes, as depicted in Fig. 3. Mainly, dabigatran presents a significantly lower mortality risk than the other three DOACs. In contrast, low-dose rivaroxaban, apixaban, and edoxaban were associated with increased mortality risks, with hazard ratios of 1.71 (95% CI 1.05–2.77, *p* = 0.030), 2.21 (95% CI 1.27–3.83, *p* = 0.005), and 2.18 (95% CI 1.22–3.89, *p* = 0.008), respectively. These findings were starkly contrasted to those observed with standard-dose dabigatran, which showed no statistically significant differences from the other DOACs. Furthermore, in an investigation of individual DOACs (Fig. 4), no significant difference in mortality rates was observed between standard and low-dose regimens.

### Management of major bleeding

Among the 2770 patients who experienced DOAC-related major bleeding events, 11.3% underwent surgical intervention, while 35.3% received medical interventions such as endoscopic hemostasis or transarterial embolization (TAE). Additionally, 7.9% required blood transfusions of more than four units, as detailed in Table 4. Patients treated with dabigatran were more likely to receive endoscopic hemostasis, TAE, and blood transfusions. Notably, the differences in medical intervention and blood transfusion rates were statistically significant when comparing the dabigatran group with the apixaban group.

**Table 4:** Management of major bleeding and transfusion volumes at different bleeding sites in patients treated with direct oral anticoagulants.

	Total					
	(n = 2770)	Dabigatran (n = 460)	Rivaroxaban (n = 1322)	Apixaban (n = 548)	Edoxaban (n = 440)	*p* value^a^
Surgical intervention	11.3%	11.1%	11.9%	11.9%	9.3%	0.5040
Endoscopic/TAE intervention	35.3%	41.5%	33.9%	31.4%*	37.7%	0.0094
Blood transfusion (> 4 units)	7.9%	10.2%	7.1%	6.4%*	10.0%	0.0311
ICH	787 (28.4%)	91 (19.8%)	420 (31.8%)	172 (31.4%)	104 (23.6%)	
Blood transfusion (> 4 units)	3.8%	2.2%	4.0%	4.3%	3.9%	0.8633
GI	1671 (60.3%)	323 (70.2%)	757 (57.3%)	310 (56.6%)	281 (63.9%)	
Blood transfusion (> 4 units)	10.7%	13.6%	9.5%	8.1%*	13.4%	0.0352
Other critical sites	346 (12.5%)	50 (10.9%)	161 (12.2%)	74 (13.5%)	61 (13.9%)	
Blood transfusion (> 4 units)	10.1%	21.3%	7.9%*	7.1%*	10.2%	0.0238

TAE, transarterial embolization; ICH, intracranial haemorrhage; GI, gastrointestinal.

^a^Chi-square test.

*Reach statistical significance, when compared with the dabigatran group.

Of these 2770 patients, 60.3% experienced GI bleeding, 28.4% ICH, and the remaining 12.5% suffered bleeding in other sites. The incidence of ICH as a major bleeding type was 19.8% in the dabigatran group, 31.8% in the rivaroxaban group, 31.4% in the apixaban group, and 23.6% in the edoxaban group. The dabigatran group exhibited a higher proportion of GI bleeding (70.2%), followed by edoxaban (63.9%), rivaroxaban (57.3%), and apixaban (56.6%). Notably, a significantly higher proportion in the dabigatran group needed blood transfusions (> 4 units) for GI and other sites bleeding compared to the apixaban group. Similarly, for other site bleeding events, the need for blood transfusions (> 4 units) was significantly higher in the dabigatran group than the rivaroxaban group.

## Discussion

In this nationwide retrospective cohort study of 2770 major bleeding events among Asian AF patients treated with DOACs, we found that the hazard ratio for 7-day mortality in those treated with factor Xa inhibitors was twice those treated with dabigatran. Additionally, the 7-day mortality rate was lower following dabigatran bleeding events than factor Xa inhibitors, although the differences in 30-day mortality did not reach statistical significance. Furthermore, the initial 7-day mortality rates varied between 4.6 and 11.1% among different DOACs, but decreased to 7.0% in the 8–30 day period after the bleeding event. This finding underscores the importance of prompt identification and expedited management, particularly during the first week after a major bleeding event.

The mortality rate after major bleeding events in our study involving DOACs aligns with previous research^16,17^. In our study, the 30-day mortality rate related to major bleeding was 16%, which is consistent with a cohort study that included 2002 patients presenting to the hospital with DOAC or warfarin-related bleeding, reporting a 30-day mortality rate of 12.6% for DOAC-related major bleeding^17^. In our research, the 7-day and 30-day mortality rates in patients receiving dabigatran were 4.6% and 11.5%, respectively. These rates are compatible with a phase III trial that indicated a 7-day and 30-day mortality rate of 5.3% and 9.1% for dabigatran^16^. Although the consistency of these findings across studies is noteworthy, there remains a gap in the research regarding direct comparisons among different DOACs. As a result, our study fills this gap by highlighting differences in the 7-day mortality rate among DOACs. Specifically, we observed that Factor Xa inhibitors had a mortality rate of 9.2–11.1%, twice of dabigatran (4.6%).

NOACs-related ICH is the most feared major bleeding event due to its associated morbidity and mortality. In our study, the 7-day ICH-related mortality rate for NOACs ranged from 12.0 to 29.8%, which is higher than the GI-bleeding-related mortality rate of 2.79–5.48%. Among different types of bleeding events, ICH accounted for 28.4% of cases but accounted for nearly half of the deaths. This finding aligns with a multinational study where ICH represented 24.8% of patients and was identified as the primary cause of death within one month following a major bleeding event with NOACs^8^. Of note, the dabigatran group showed a lower ICH incidence (19.8%) compared to factor Xa inhibitors (23.6–31.8%), suggesting a potential link between lower ICH rates and reduced 7-day mortality. In contrast to ICH, GI bleeding usually leads to earlier medical intervention. The dabigatran group not only experienced more GI bleeding events (70.2%) compared to factor Xa inhibitors (56.6%-63.9%) but also underwent more medical interventions (41.5%). This proactive approach in treating GI bleeding may explain the lower 7-day mortality seen in the dabigatran group after major bleeding events.

The physiological mechanisms underlying the lower 7-day mortality of dabigatran compared to Xa inhibitors remain unclear. However, one potential explanation could be attributed to inherent differences between dabigatran and Xa inhibitors. For instance, in a study involving 9769 patients with nonvalvular atrial fibrillation, dabigatran was found to be associated with a lower risk of acute kidney injury (AKI) when compared to warfarin^25^. In contrast, the Xa inhibitor apixaban did not demonstrate a statistically significant relationship with renal outcomes. It is worth noting that AKI is closely linked to mortality outcomes in patients experiencing bleeding events^26,27^. Therefore, we cannot exclude the possibility of different AKI outcomes among NOACs contributing to the varying mortality rates after major bleeding. Further research is needed to elucidate the underlying mechanisms.

Previous studies demonstrated that even low-dose DOACs have a favorable safety profile compared to warfarin in patients with atrial fibrillation^3^. The use of low-dose DOACs in treating AF becomes particularly notable in East Asia, where specific factors such as a lower average body mass index, ethnicity, and renal clearance of DOACs have influenced the preference for prescribing low-dose DOACs^28,29^. This trend is evident in nationwide cohorts from Taiwan and Korea, where nearly 90% and more than 60% of patients are prescribed low-dose dabigatran and rivaroxaban, respectively^30,31^. Of note, in our study including patients with NOACs-related major bleeding, there was also a higher prevalence of low-dose use of DOACs than standard-dose. Even though, low-dose regimens of four DOACs exhibit considerable disparities in outcomes. Dabigatran still presents a significantly lower mortality risk than the other three DOACs of Xa inhibitors.

This study stands out for its robust methodology and significant insights into the short-term mortality outcomes associated with different DOACs in Asian AF patients. The use of the Taiwan NHIRD, which covers 99% of the Taiwanese population, enabled a large-scale, nationwide retrospective cohort study. The study's propensity score stabilized weighting ensures well-balanced comparison groups to enhance the validity of our findings. Furthermore, our focus on both 7-day and 30-day mortality provides a nuanced understanding of the immediate and short-term risks following major bleeding events. The subgroup analyses across different bleeding sites and dose regimens offer valuable insights, especially in the context of East Asian patient populations where low-dose DOACs are prevalently prescribed. By accessing the death registry system, we demonstrated the cause of death, categorized by the specific type of oral anticoagulant administered. Our findings fill a critical gap in existing literature by comparing short-term mortality outcomes among all four major DOACs, providing crucial information for clinicians in managing AF patients at risk of major bleeding.Our study has some limitations. First, our study lacks information about post-bleeding management, such as administration of prothrombin complex concentrates, which may have an impact on clinical outcomes after bleeding. Second, there were only a small number of patients (n = 20) who received the reversal agent idarucizumab during the study period. Additionally, the reversal agent for factor Xa inhibitors was not available in Taiwan. Therefore, we excluded patients who received idarucizumab for the reversal of dabigatran effect in our study. Third, the NHIRD does not capture information such as creatinine level or body weight data, which may affect the clinical mortality outcomes.

In conclusion, our study found a higher hazard ratio for 7-day mortality in Asian AF patients with major bleeding related to Factor Xa inhibitors than dabigatran, although differences in 30-day mortality did not reach statistical significance. The mortality rate mainly occurred within the initial 7-day period, emphasizing the urgent need for prompt identification and intervention in cases of major bleeding.

### Supplementary Information

Supplementary Tables.

## Data Availability

The data for this study were sourced from the National Health Insurance Research Database Taiwan (NHIRD), which is housed exclusively in the Health and Welfare Data Science Center. Due to restrictions on data sharing, we are unable to make the research data publicly available. However, the datasets used and/or analyzed in this study can be made available by the corresponding author upon reasonable request.
