# Supplementary material for: Mortality after major bleeding in Asian atrial fibrillation patients receiving different direct oral anticoagulants: a nationwide, propensity score study
Source: Sci Rep. 2024 Feb 27;14:4771. doi: 10.1038/s41598-024-55500-z (PMC10899247; doi:10.1038/s41598-024-55500-z)
Supplement: Supplementary file 1 — Supplementary Tables. [file 41598_2024_55500_MOESM1_ESM.pdf]

# Mortality after Major Bleeding in Asian Atrial Fibrillation Patients Receiving Different Direct Oral Anticoagulants: A Nationwide, Propensity Score Study

Jiun-Hao Yu, Pei-Ru Li, Dong-Yi Chen, Wen-Kuan Huang, and Lai-Chu See

**Supplementary Table S1.** International Classification of Diseases (Ninth and Tenth Revisions) Clinical Modification (ICD-9-CM and ICD-10-CM) codes used to define major bleeding and comorbidities

| Disease | ICD-9-CM Codes | ICD-10-CM Codes | Diagnosis definition |
|---|---|---|---|
| (A) Major bleeding | | | |
| Intracranial hemorrhage | 430, 431, 432, 852, 853, 804, 803, 801, 800 | I60, I61, I62, S06.6X0A, S06.6X9A, S06.5X0A, S06.5X9A, S06.4X0A, S06.4X9A, S06.9X0A, S06.9X9A | Patient enrolled: emergency department; history: discharge |
| Gastrointestinal bleeding | 456.0, 456.2, 455.2, 455.5, 455.8, 530.7, 530.82, 531.0 – 531.6, 532.0 – 532.6, 533.0 – 533.6, 534.0 – 534.6, 535.0 – 535.6 537.83, 562.02, 562.03, 562.12 562.13 568.81, 569.3, 569.85, 578.0, 578.1, 578.9 | K22.6, K25.0, K25.2, K25.4, K25.6, K26.0, K26.2, K26.4, K26.6, K27.0, K27.2, K27.4, K27.6, K28.0, K28.2, K28.4, K28.6, K29.0, K62.5, K92.0, K92.1, K92.2, I85.01 | Patient enrolled: emergency department; history: discharge |
| Other critical sites bleeding | 423,0, 459.0, 568.81, 593.81, 599.7, 623.8, 626.32, 626.6, 719.1, 784.7, 784.8, 786.3, 519.09, 785.59, 958.4, 860.2, 860.3, 861.21, 861.31, 862.0, 862.1, 862.21, 862.31, 862.22, 901.0, 901.40, 901.41, 901.42, 864.00, 864.10, 965.00, 902.9, 865.10 | D62, J942, H113, H356, H431, N02, N95, R04, R31, R58, I71, K66.1, J95.01, R57.1, J94.2, T79.4XXA, S27.1XXA, S27.329A, S26.01XA, S27.809A, S27.409A, S27.819A, S25.00XA, S25.409A, S36.119A, S36.00XA, S35.90XA, S36.90XA, S35.00XA, S35.10XA, S35.299A, S35.403A, S35.513A, S31.001A, T79.4XXA, S37.009A, S37.90XA, S48.019A, S48.919A, S58.019A, S58.919A, S68.419A, S78.019A, S78.919A, S88.019A, S88.119A, S98.019A | Patient enrolled: emergency department; history: discharge |
| (B) Comorbidities | | | |
| Hypertension | 401, 402 | I10-I16 | Outpatient department ≥2 |
| Hyperlipidemia | 272 | E78 | Outpatient department ≥2 |
| Ischemic stroke | 433, 434, 436 | I63, I64 | Discharge |
| Congestive heart | 428 | I11.0, I13.0, I13.2, I42.0, I50, | Discharge |

| Disease | ICD-9-CM Codes | ICD-10-CM Codes | Diagnosis definition |
|---|---|---|---|
| failure | | I50.1, I50.9 | |
| Ischemic heart disease | 410, 411, 412, 413, 414 | I21-I25 | Outpatient department ≥2 |
| Peripheral arterial disease | 440.0, 440.2, 440.3, 440.8, 440.9, 443, 444.0, 444.22, 444.8, 444.9, 447.9, 440.0, 38.08, 38.18, 38.38, 38.48, 38.68, 38.88, 39.50, 39.7, 39.90, 39.25, 39.26, 39.29, 84.10-84.15, 84.16-84.19 | I70.0, I70.2, I70.9, I70.3, I70.8, I75.89, I70.9, I73.0, I73.1, I73.8, I73.9, I79.1, I79.8, I74.01, I74.09, I74.3, I74.4, I74.5, I74.8, I74.9, I77.9, I70.0, 41, 045, 047, 049, 04B, 04C, 04H, 04J, 04L, 04N, 04P, 04Q, 04R, 04S, 04U, 04V, 04W Location:(C,D,E,F,H,J,K,L,M,N,P,Q, R,S,T,U,V,W,Y)<br>0Y67, 0Y68, 0Y6C, 0Y6D, 0Y6F, 0Y6G, 0Y6H, 0Y6J, 0Y6M, 0Y6N, 0Y6P, 0Y6Q, 0Y6R, 0Y6S, 0Y6T, 0Y6U, 0Y6V, 0Y6W, 0Y6X, 0Y6Y | Discharge or Outpatient department ≥2 |
| Diabetes mellitus | 250 | E11, E13 | Outpatient department ≥2 |
| Chronic kidney disease | 580-589 | I12, I13, N00, N01, N02, N03, N04, N05, N07, N11, N14, N17, N18, N19, Q61 | Outpatient department ≥2 |
| Chronic lung disease | 490, 491.0, 491.1, 491.20-491.22, 491.8, 491.9, 492.0, 492.8, 493.00-493.02 493.10-493.12, 493.20-493.22, 493.81, 493.82, 493.90-493.92, 494.0, 494.1, 495.8, 495.9, 496, 500, 502, 503, 504, 505 | J41-J44 | Discharge |
| Chronic liver disease | 570, 571, 572 | B150, B160, B162, B190, K704, K72, K766, I85 | Outpatient department ≥2 |
| Malignancy | 140.0-208.9 | C | Outpatient department ≥2 |
| Transient ischemic attack | 435 | G45 | Discharge |

Noted: ICD-9-CM codes were used during 2000–2015, and ICD-10-CM codes were used after 2016.

**Supplementary Table S2.** Surgical and Medical Hemostasis Approaches in Patients with Major Bleeding

| **Surgical Intervention** | |
|---|---|
| Brain surgery | • Burr hole (trephination) for hemostasis<br>• Craniectomy<br>• Removal of epidural hematoma<br>• Removal of acute subdural hematoma<br>• Removal of chronic subdural hematoma<br>• Removal of intracerebral hematoma<br>• Intracranial pressure monitoring<br>• Craniotomy for vascular lesions<br>• Excision of intraspinal arteriovenous malformation<br>• Ventricular-peritoneal shunt<br>• Operation for depressed fracture of skull |
| Surgery for Other Sites | • Exploratory laparotomy<br>• Hepatorrhaphy, suture of liver wound<br>• Lobectomy<br>• Partial hepatectomy<br>• Repair of intestinal perforation<br>• Splenectomy<br>• Exploration, vascular<br>• Pericardiotomy with exploration<br>• Pericardial puncture<br>• Pericardiocentesis<br>• Exploratory thoracotomy<br>• Nephrectomy<br>• Partial nephrectomy<br>• Surgery for posterior epistaxis |
| **Medical Intervention** | |
| Endoscopic Hemostasis | • Upper gastrointestinal panendoscopy<br>• Colonoscopy<br>• Bronchoscopy<br>• Enteroscopy |

| | |
|---|---|
| | • Rectoscopy<br>• Sigmoidoscopy<br>• Laparoscopy<br>• Esophageal endoscopy<br>• Endoscopic control of hemorrhage, rectum and/or fulguration<br>• Endoscopic hemostasis for colon bleeding |
| Other medical intervention | • Trans-arterial embolization<br>• Gastrointestinal bleeding embolisation |

**Supplementary Table S3.** Demographic characteristics and comorbidities when admitted to emergency department due to major bleeding among patients with atrial fibrillation and treated with the four direct oral anticoagulants in Taiwan, 2016-2019, before propensity score stabilized weighting

| | **Total (n=2770)** | **Dabigatran (n=460)** | **Rivaroxaban (n=1322)** | **Apixaban (n=548)** | **Edoxaban (n=440)** | **max ASMD** |
|---|---|---|---|---|---|---|
| Age, years | 79.6±10.0 | 80.2±9.6 | 78.9±10.2 | 79.8±10.4 | 80.9±9.4 | 0.199 |
| Male | 1486 (53.6%) | 246 (53.5%) | 705 (53.3%) | 301 (54.9%) | 234 (53.2%) | 0.035 |
| $CHA_2DS_2$-VASc | 4.8±1.8 | 4.6±1.7 | 4.8±1.8 | 4.9±1.8 | 4.9±1.9 | 0.1408 |
| HAS-BLED | 3.6±1.3 | 3.5±1.2 | 3.6±1.3 | 3.8±1.3 | 3.7±1.3 | 0.1271 |
| History of bleeding | 1634 (59.0%) | 276 (60.0%) | 776 (58.7%) | 327 (59.7%) | 255 (58.0%) | 0.0415 |
| Hypertension | 1957 (70.6%) | 322 (70.0%) | 917 (69.4%) | 397 (72.5%) | 321 (73.0%) | 0.0792 |
| Hyperlipidemia | 1159 (41.8%) | 174 (37.8%) | 561 (42.4%) | 238 (43.4%) | 186 (42.3%) | 0.1142 |
| Cardiovascular disease | 1826 (65.9%) | 294 (63.9%) | 861 (65.1%) | 385 (70.3%) | 286 (65.0%) | 0.1351 |
| Stroke | 1244 (44.9%) | 175 (38.0%) | 619 (46.8%) | 277 (50.6%) | 173 (39.3%) | |
| Congestive heart failure | 506 (18.3%) | 100 (21.7%) | 200 (15.1%) | 112 (20.4%) | 94 (21.4%) | |
| Chronic ischemic heart disease | 499 (18.0%) | 83 (18.0%) | 244 (18.5%) | 78 (14.2%) | 94 (21.4%) | |
| Peripheral arterial disease | 46 (1.7%) | 5 (1.1%) | 29 (2.2%) | 7 (1.3%) | 5 (1.1%) | |
| Coronary artery bypass graft | 51 (1.8%) | 7 (1.5%) | 23 (1.7%) | 11 (2.0%) | 10 (2.3%) | |
| Percutaneous coronary intervention | 304 (11.0%) | 46 (10.0%) | 149 (11.3%) | 48 (8.8%) | 61 (13.9%) | |
| Diabetes mellitus | 1148 (41.4%) | 177 (38.5%) | 550 (41.6%) | 241 (44.0%) | 180 (40.9%) | 0.1118 |
| Chronic kidney disease | 688 (24.8%) | 114 (24.8%) | 304 (23.0%) | 152 (27.7%) | 118 (26.8%) | 0.1091 |
| Chronic lung disease | 448 (16.2%) | 60 (13.0%) | 205 (15.5%) | 92 (16.8%) | 91 (20.7%) | 0.2048 |
| Chronic liver disease | 218 (7.9%) | 38 (8.3%) | 106 (8.0%) | 39 (7.1%) | 35 (8.0%) | 0.0429 |
| Malignancy | 598 (21.6%) | 75 (16.3%) | 298 (22.5%) | 131 (23.9%) | 94 (21.4%) | 0.1903 |
| Use of antiplatelet | 625 (22.6%) | 106 (23.0%) | 287 (21.7%) | 123 (22.4%) | 109 (24.8%) | 0.0548 |
| Use of NSAIDs | 879 (31.7%) | 157 (34.1%) | 428 (32.4%) | 179 (32.7%) | 115 (26.1%) | 0.1747 |
| Use of PPI | 1608 (58.1%) | 301 (65.4%) | 734 (55.5%) | 312 (56.9%) | 261 (59.3%) | 0.2037 |
| Use of ACEI/ARB | 1758 (63.5%) | 290 (63.0%) | 850 (64.3%) | 343 (62.6%) | 275 (62.5%) | 0.0373 |
| Use of β-blocker | 1610 (58.1%) | 274 (59.6%) | 759 (57.4%) | 310 (56.6%) | 267 (60.7%) | 0.0835 |
| Use of dihydropyridine CCB | 1403 (50.6%) | 208 (45.2%) | 672 (50.8%) | 298 (54.4%) | 225 (51.1%) | 0.1838 |
| Use of diltiazem/verapamil | 618 (22.3%) | 113 (24.6%) | 285 (21.6%) | 130 (23.7%) | 90 (20.5%) | 0.0984 |
| Use of statin | 970 (35.0%) | 149 (32.4%) | 466 (35.2%) | 200 (36.5%) | 145 (33.0%) | 0.0864 |
| Use of digoxin | 490 (17.7%) | 91 (19.8%) | 219 (16.6%) | 92 (16.8%) | 88 (20.0%) | 0.0889 |

ACEI indicates angiotensin-converting enzyme inhibitor; ARB, angiotensin II receptor antagonists; $CHA_2DS_2$-VASc, congestive heart failure, hypertension, age 75 years or older, diabetes mellitus, stroke/transient ischemic attack, vascular disease, age 65 to 74 years, female; CCB, calcium channel blockers; HAS-BLED, hypertension, abnormal renal or liver function, stroke, bleeding history, age 65 years or older, and antiplatelet drug use; NSAIDs, nonsteroidal anti-inflammatory drugs; PPI, proton pump inhibitor.
